# Bridging Awareness to Community Engagement on Indoor Air Pollution: A Pilot Health Education Activity in an Urban Slum of Bhopal, India

**DOI:** 10.7759/cureus.108068

**Published:** 2026-04-30

**Authors:** Suparna Mishra, Sanskriti Kalura, Aakanksha Patil

**Affiliations:** 1 Community and Family Medicine, All India Institute of Medical Sciences, Bhopal, Bhopal, IND

**Keywords:** health promotion, iec materials, indoor air pollution, knowledge attitudes and practices, smog readability index

## Abstract

Background

Indoor air pollution (IAP) is a significant public health problem in urban slum settings in India. Even in households using liquefied petroleum gas (LPG), exposure to indoor pollutants continues due to poor ventilation, supplementary solid fuel use, and burning of incense and mosquito coils. Most available information, education, and communication (IEC) materials on air pollution address outdoor pollution and are not suited to household-level exposures.

Methods

A community-based health promotion activity was carried out in an urban slum of Ward 49, Bhopal, India, from May to June 2025. It had four phases: (1) a household-level knowledge, attitude, and practice (KAP) survey (n=32); (2) a review of existing IEC materials on air pollution; (3) development and community-based refinement of Hindi-language IEC posters, assessed using the Simple Measure of Gobbledygook (SMOG) readability index and the teach-back method; and (4) pilot implementation through health talks at the Urban Primary Health Centre (UPHC).

Results

Most respondents were women (78%), with a mean age of 42 years. All households used LPG as their primary cooking fuel, but 56% (18/32) also used supplementary solid fuels. Nearly two-thirds of households (62.5%, 20/32) had never heard of IAP as a concept; awareness of pollution sources was limited mostly to outdoor vehicle emissions. Practice gaps included no chimney or exhaust fan in 94% of cooking areas, cooking in the living room in 56% of households, and daily use of incense sticks or mosquito coils in 75% of respondents. The revised IEC poster had an SMOG grade of 8.1, compared to 10.2 for an existing national IEC poster, reflecting better readability. Health talks were held at the UPHC, and IEC materials were distributed.

Conclusion

A structured, community needs-based approach to IAP health communication is feasible in urban slum settings. IEC materials developed from local assessments are more readable and relevant and may help support behaviour change in households transitioning away from solid fuels.

## Introduction

Indoor air pollution (IAP) is one of the leading environmental causes of premature death worldwide. The World Health Organization estimates that IAP caused approximately 3.2 million deaths globally in 2019, with the largest burden in low- and middle-income countries (LMICs) [1]. In India, the Global Burden of Disease Study 2019 attributed around 0.61 million deaths to household air pollution in that year [2]. The Pradhan Mantri Ujjwala Yojana (PMUY) has greatly expanded liquefied petroleum gas (LPG) access among low-income households [3], but IAP has not gone away. Despite this progress, IAP remains a significant concern in India. Studies in urban slums have documented indoor PM2.5 levels substantially exceeding WHO guidelines - with measurements in Mumbai slums reaching two to four times the national annual standard of 40 µg/m³, particularly in homes with poor ventilation and those reporting mosquito coil use [4]. Nationally, household air pollution contributed to approximately 0.61 million deaths in India in 2019, accounting for nearly 18% of total deaths in the country [2]. Supplementary solid fuel use, poor ventilation, and daily burning of incense sticks and mosquito coils continue to keep indoor pollutant levels high even in households that have switched to LPG.

Urban slums in India add another layer of difficulty. Dense housing, no separate kitchen, windows only at the front of the house while cooking happens at the back, and no exhaust fans, all of these mean that pollutants build up indoors regardless of what fuel is used [4]. However, awareness of IAP as a health risk is low among slum residents [5,6]. Most people associate air pollution with vehicle smoke outdoors and do not see indoor practices such as burning incense or using a chulha occasionally as a problem [5,6]. Importantly, many structural contributors to IAP - such as the absence of exhaust infrastructure and the placement of cooking areas away from windows - cannot be addressed through education alone, underscoring the need for an IEC that focuses on immediately feasible, low-cost behavioural actions rather than structural modifications.

IEC materials are a basic tool of community health promotion. A review of national IEC resources on air pollution shows a clear gap: nearly all materials focus on outdoor or ambient pollution and say little about household-level IAP. Many are also written in English or difficult Hindi, which makes them hard to use in semi-literate communities - the Simple Measure of Gobbledygook (SMOG) readability index, first described by McLaughlin in 1969 [7], and the teach-back method, widely used to check and improve health communication with low-literacy audiences [8], offer practical tools to address this gap. The activity was grounded in the health belief model and self-efficacy theory, which informed the focus on low-cost, immediately actionable behaviour change messages that participants would feel capable of adopting.

This activity was carried out in an urban slum of Bhopal with the following objectives: (1) to identify gaps in knowledge and household practices that contribute to indoor air pollution in the study community; (2) to sensitise community members about IAP and its health effects; (3) to promote simple, feasible behaviour changes that can reduce household IAP exposure; and (4) to develop, refine, and distribute locally relevant, easy-to-understand Hindi-language IEC materials for community use [8].

## Materials and methods

Study design and setting

This was a community-based descriptive health promotion study with a cross-sectional knowledge, attitude, and practice (KAP) survey component, conducted in the urban slum of Ward 49, Bhopal, Madhya Pradesh, India. The area falls within the field practice area of the Department of Community and Family Medicine, All India Institute of Medical Sciences (AIIMS) Bhopal. The study was conducted from May to June 2025 and had four phases, described below.

Phase 1: KAP Survey

A household-level KAP survey was done to assess knowledge about IAP, household practices contributing to indoor pollution, and barriers to behaviour change. Households were approached through a systematic field-based convenience approach during field visits starting from a fixed landmark - the entry point of the main lane in the locality - with each subsequent household being the next accessible dwelling along the lane. If a household was locked or no eligible adult was available, the next household in sequence was approached. Convenience sampling was used as this was a formative pilot activity and not a hypothesis-testing study; formal sample size calculation was therefore not applicable. The target of 35 households was determined pragmatically based on the number of households accessible within the field practice area during the available study period. One adult member from each consenting household was selected for interview using the Kish grid method [9], which gives each eligible adult an equal chance of being selected. Interviews were conducted face-to-face in Hindi using a structured questionnaire.

Participants had to be 18 years or older, living in the household for at least six months, and present at the time of the visit. Households with a shop or business inside the home were excluded, as these settings may harbour atypical pollutant sources unrepresentative of domestic IAP exposure. Households with temporary residents were excluded to ensure that reported practices reflected established household behaviour.

The questionnaire had two parts. The first collected sociodemographic details - age, sex, education, occupation, monthly income, family type, and housing type. The second part had 14 items on household practices related to IAP, awareness about air pollution, and perceived barriers to improving ventilation or reducing indoor pollutant exposures. The questionnaire was developed de novo for this activity, as no validated standardised tool for household IAP assessment in the Indian urban slum context was available [5,6]. The tool was pilot tested with seven individuals from a similar nearby community and refined based on feedback from faculty and senior residents of the department.

Phase 2: Review of Existing IEC Materials

**Figure 1:**
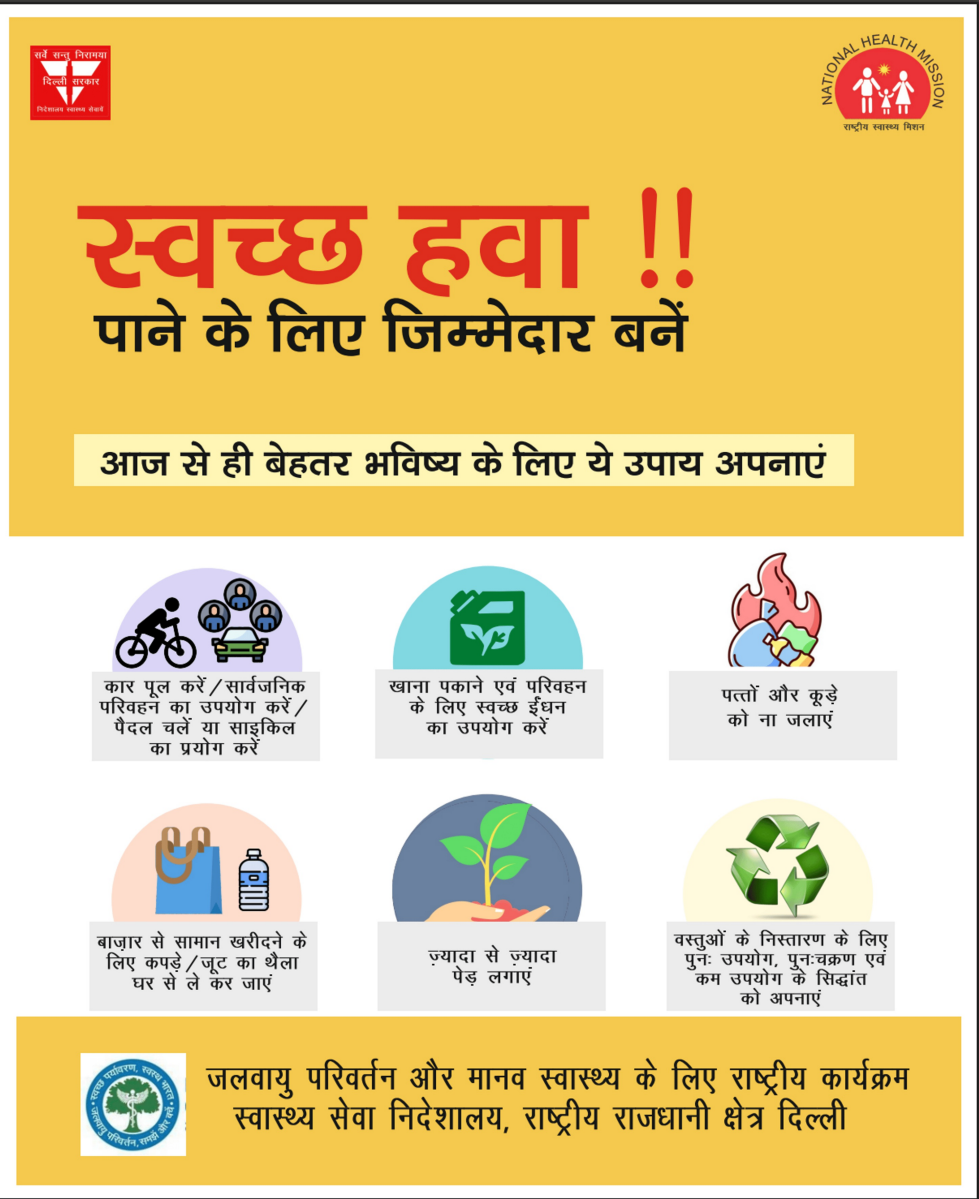
Existing IEC materials on air pollution from national government sources Poster from the Directorate General of Health Services (DGHS), Delhi, under the National Programme on Climate Change and Human Health [10]. All six recommended actions address outdoor behaviour (carpooling, cycling, tree planting, waste burning avoidance, recycling). No mention of indoor air pollution sources. IEC: information, education, and communication Source: Directorate General of Health Services, Government of NCT of Delhi (https://dgehs.delhi.gov.in/); image in the public domain

**Figure 2:**
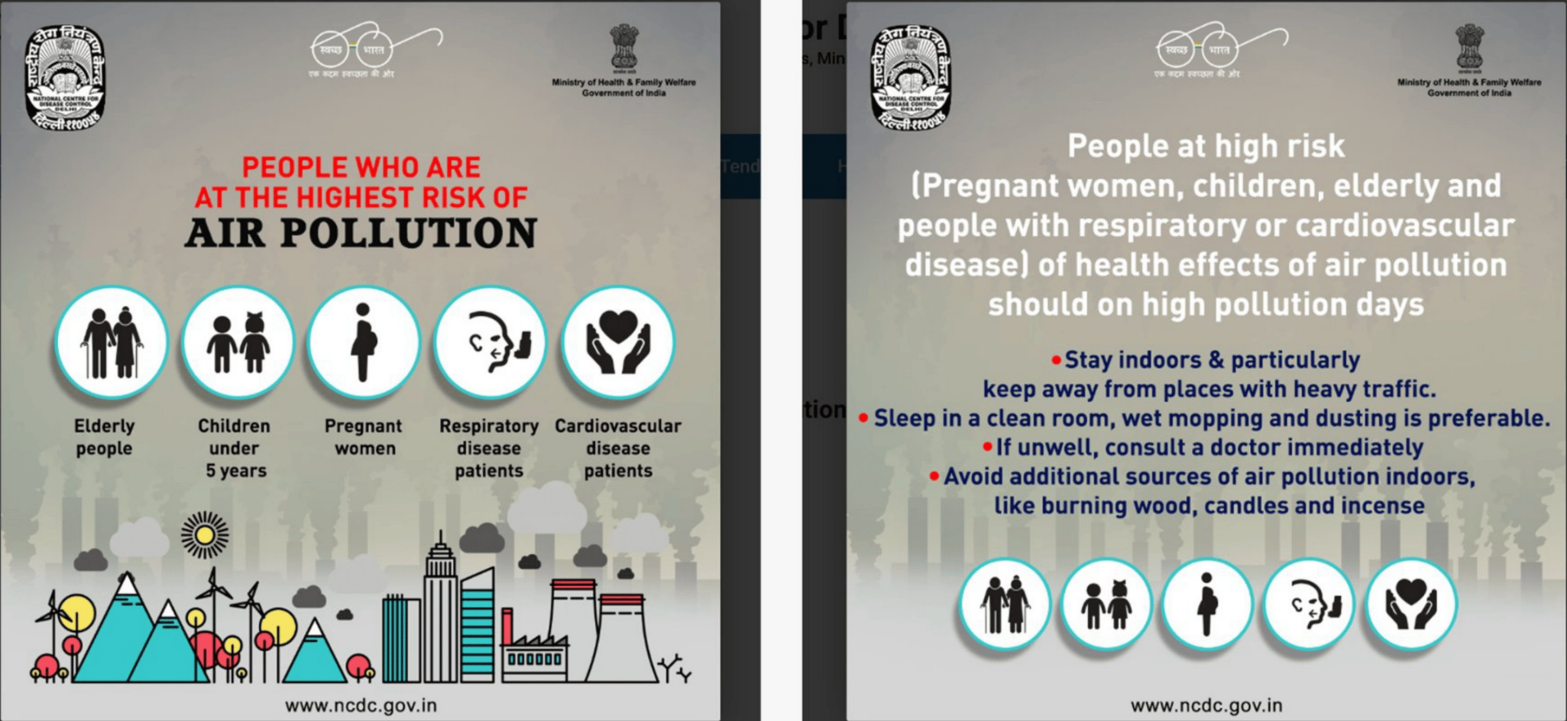
Existing IEC materials on air pollution from national government sources Posters from the National Centre for Disease Control (NCDC), Ministry of Health and Family Welfare, Government of India [10]. High-risk groups are correctly identified, but air pollution is framed entirely in the outdoor ambient context. Language is English only. SMOG grade: 10.2. Source: Directorate General of Health Services, Government of NCT of Delhi (https://dgehs.delhi.gov.in/); image in the public domain IEC: information, education, and communication; SMOG: Simple Measure of Gobbledygook

A structured document review was conducted between April and May 2025 to identify IEC materials on air pollution from national government sources (Figures 1-2) - the National Clean Air Programme (NCAP) [10], the Ministry of Health and Family Welfare, the National Centre for Disease Control (NCDC), and the Directorate General of Health Services (DGHS), Delhi. Each material was assessed for its relevance to indoor air pollution, language and literacy level, visual clarity, and whether it included practical household-level advice. Readability of English-language materials was measured using the SMOG index [7]. Readability assessment is particularly important in low-literacy settings, such as urban slums, where materials written above the community's reading level are unlikely to be understood or acted upon, regardless of their scientific accuracy or visual quality [8].

The Bhopal City Action Plan under NCAP [10] lists public awareness activities - including issuance of advisories and school-based programmes - but no specific IEC materials for household-level IAP were available from this source. None of the reviewed materials addressed household-level IAP sources, such as incense sticks, mosquito coils, solid fuel cooking, or poor ventilation - all focused exclusively on outdoor or ambient air pollution, including vehicle emissions, industrial sources, and crop burning. No material was available in simple, locally contextualised Hindi. A summary of the reviewed materials and their readability is shown in Table 1. 

**Table 1:** Summary of existing IEC materials reviewed and SMOG readability comparison A lower SMOG score indicates greater readability. IAP: indoor air pollution; IEC: information, education, and communication; NCDC: National Centre for Disease Control; DGHS: Directorate General of Health Services; NPCCHH: National Programme on Climate Change and Human Health; NCAP: National Clean Air Programme; SMOG: Simple Measure of Gobbledygook

Source	Language	Topic focus	Household IAP addressed
NCDC poster (Ministry of Health & Family Welfare)	English	Outdoor/ambient air pollution - high-risk groups and behavioural advice for high pollution days	No
DGHS Delhi/NPCCHH poster	Hindi	Outdoor pollution - vehicle use, waste burning avoidance, tree planting, recycling	No
NCAP Bhopal City Action Plan	English	General public awareness activities; advisory issuance and school programmes listed	No
Study poster - initial draft	Hindi	Indoor sources of IAP, health effects, behaviour change messages	Yes
Study poster - final revised version	Hindi	Indoor sources of IAP, health effects, behaviour change messages	Yes

Phase 3: Development and Refinement of IEC Materials

Based on the KAP survey findings and the document review, two Hindi-language poster-based IEC materials were developed - one on common sources of IAP and their health effects and one on practical steps to reduce indoor pollution. The design was guided by two frameworks: the Health Belief Model (HBM) [11], which helped address gaps in perceived susceptibility and perceived barriers, and the Ottawa Charter for Health Promotion [12], specifically the goals of building personal skills and creating supportive environments. Specifically, perceived susceptibility was addressed by identifying vulnerable groups with pictorial icons; perceived barriers were addressed by focusing exclusively on low-cost, immediately feasible behaviour changes; and self-efficacy was reinforced through the ✗/✓ icon format, which presents each harmful practice alongside a simple, achievable alternative.

**Figure 3:**
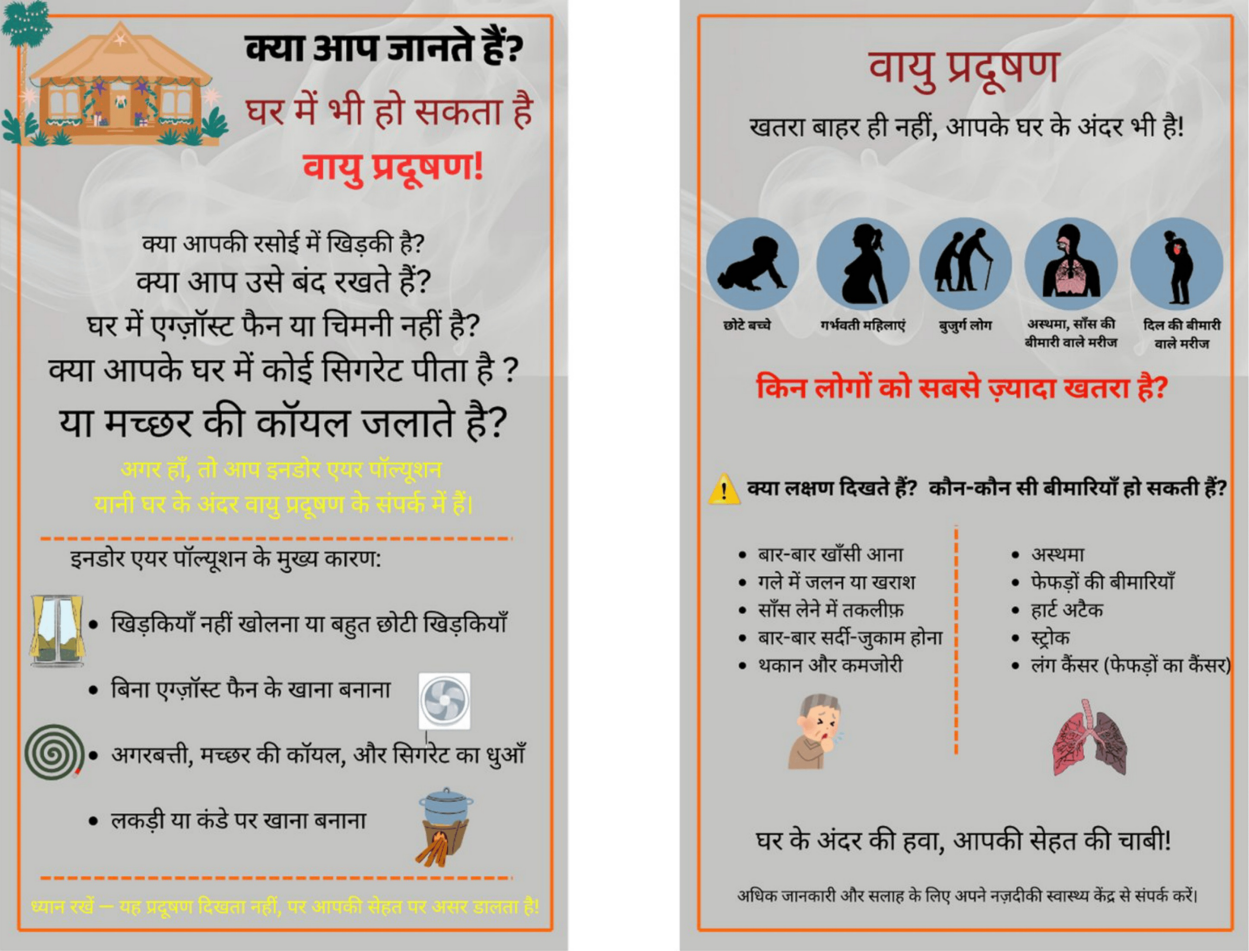
Development of the study IEC posters: initial drafts Initial draft of both IEC posters. Poster 1 (left) covers sources of indoor air pollution using a question-and-answer format and bullet points. Poster 2 (right) covers vulnerable groups and health effects. Community members reported difficulty reading the smaller text and identified limited pictorial content as a barrier to understanding. IEC: information, education, and communication

**Figure 4:**
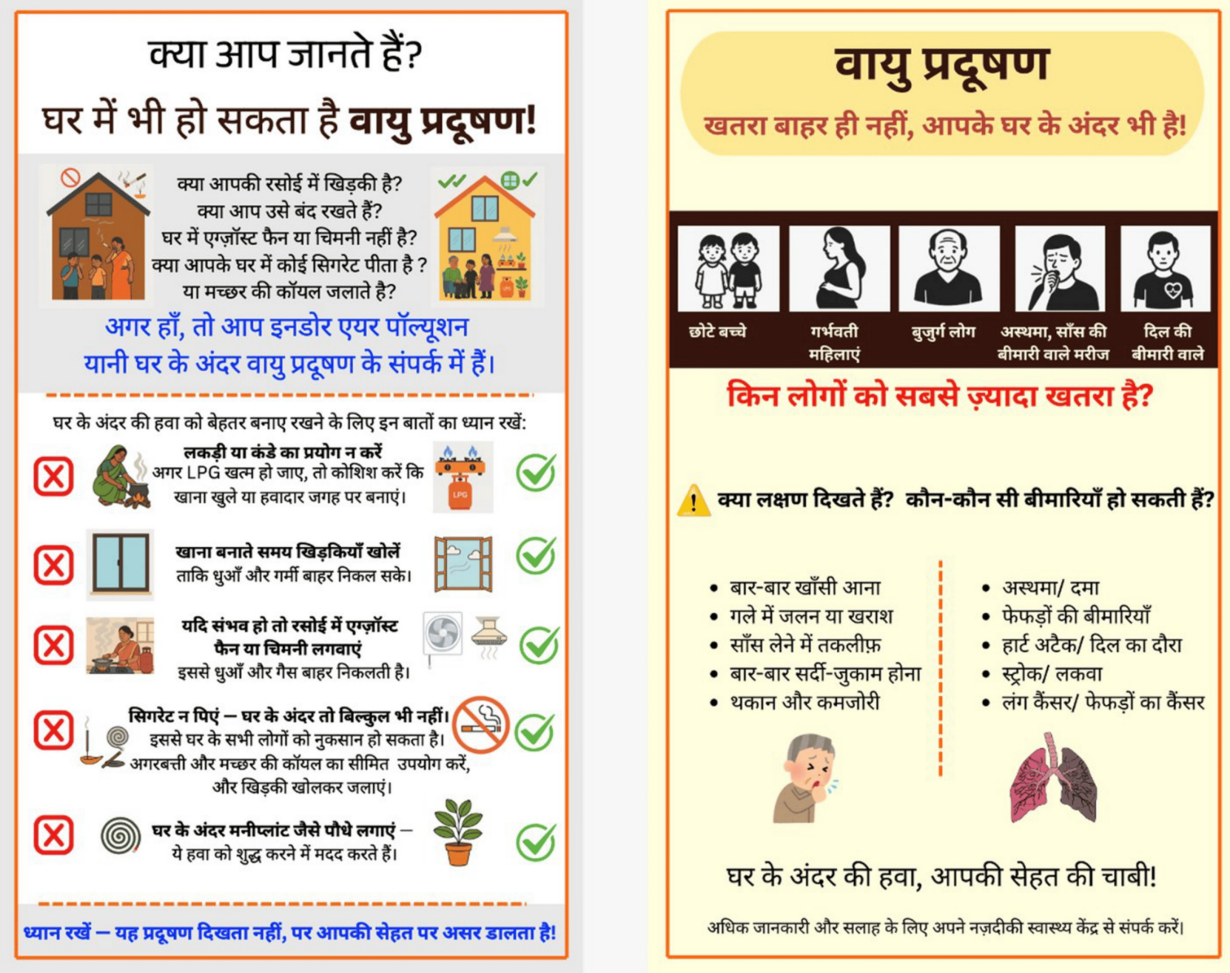
Development of the study IEC posters: intermediate revised version (after first round of community feedback) Second version following the first round of teach-back feedback. Key changes: addition of ✗/✓ icon pairs to distinguish harmful from recommended practices; pictograms added alongside each behaviour change message; font size increased; actionable recommendations framed positively (what to do, not only what to avoid). IEC: information, education, and communication

**Figure 5:**
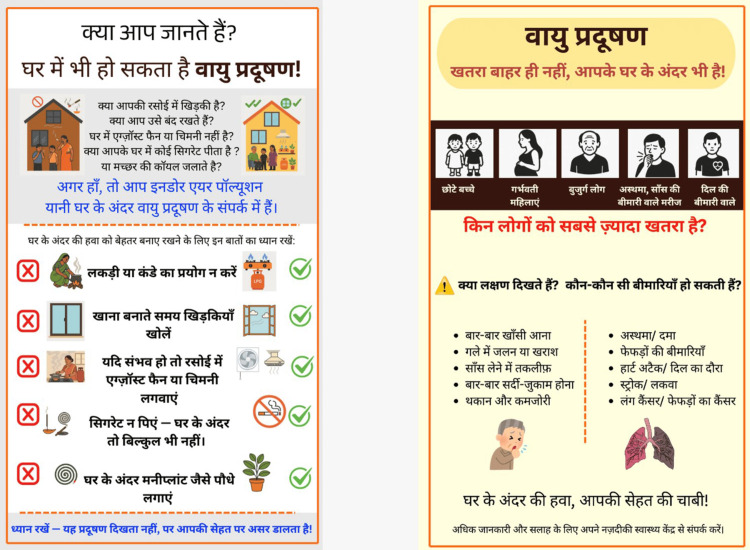
Development of the study IEC posters: final revised version Final version of both IEC posters after the second round of community refinement. Further changes include the following: simplified sentence structure, improved section separation and whitespace, and minimum 16-point Hindi font throughout. SMOG grade of final poster text: 8.1, compared to 10.2 for the NCDC reference material. These posters were used in Phase 4 at the UPHC. All three versions are original materials developed by the authors for this study; no permission required. IEC: information, education, and communication; NCDC: National Centre for Disease Control; SMOG: Simple Measure of Gobbledygook; UPHC: Urban Primary Health Centre

Draft posters (Figure 3; refer to Table 2 for the English translation) were shared with five community members - including both literate and semi-literate individuals - who were asked to explain the poster content back in their own words (teach-back method) [8]. Their responses guided two rounds of revision (Figures 4-5; refer to Tables 3-4 for the English translation). Common feedback included difficulty reading small text and a preference for more pictures. Readability of the poster text was checked using the SMOG index [7]. Design features chosen to improve accessibility included the following: high-contrast colour schemes (black on yellow, white on dark blue), no red-green colour combinations (to account for colour blindness), pictorial illustrations throughout, and a minimum font size of 16 points for Hindi text with clear spacing between sections. The final developed IEC is given as Figure 5.

**Table 2:** English translation of Hindi text in Figure 3 Poster 1 (left) covers sources of indoor air pollution and behaviour change messages. Poster 2 (right) covers vulnerable groups, symptoms, and health effects. Figure 3 contains the question-based format on Poster 1 and the sources list. Poster 2 uses circular icons for vulnerable groups.

Poster 1 (left)		Poster 2 (right)	
Hindi text	English translation	Hindi text	English translation
क्या आप जानते हैं?	Did you know?	वायु प्रदूषण	Air Pollution
घर में भी हो सकता है वायु प्रदूषण!	Air pollution can happen inside your home too!	खतरा बाहर ही नहीं, आपके घर के अंदर भी है!	The danger is not just outside — it is inside your home too!
क्या आपकी रसोई में खिड़की है?	Does your kitchen have a window?	छोटे बच्चे	Small children
क्या आप उसे बंद रखते हैं?	Do you keep it closed?	गर्भवती महिलाएं	Pregnant women
घर में एग्ज़ॉस्ट फैन या चिमनी नहीं है?	Is there no exhaust fan or chimney at home?	बुज़ुर्ग लोग	Elderly people
क्या आपके घर में कोई सिगरेट पीता है?	Does anyone in your house smoke?	अस्थमा, साँस की बीमारी वाले मरीज	Patients with asthma and respiratory diseases
या मच्छर की कॉयल जलाते है?	Or burn mosquito coils?	दिल की बीमारी वाले	People with heart disease
अगर हाँ, तो आप इनडोर एयर पॉल्यूशन यानी घर के अंदर वायु प्रदूषण के संपर्क में हैं।	If yes, you are exposed to indoor air pollution.	किन लोगों को सबसे ज़्यादा खतरा है?	Who is most at risk?
इनडोर एयर पॉल्यूशन के मुख्य कारण:	Main causes of indoor air pollution:	⚠️क्या लक्षण दिखते हैं? कौन-कौन सी बीमारियाँ हो सकती हैं?	⚠️ What symptoms appear? What diseases can occur?
• खिड़कियाँ नहीं खोलना या बहुत छोटी खिड़कियाँ	• Not opening windows or having very small windows	• बार-बार खाँसी आना	• Frequent coughing
• बिना एग्ज़ॉस्ट फैन के खाना बनाना	• Cooking without an exhaust fan	• गले में जलन या खराश	• Burning or soreness in the throat
• अगरबत्ती, मच्छर की कॉयल, और सिगरेट का धुआँ	• Smoke from incense sticks, mosquito coils, and cigarettes	• साँस लेने में तकलीफ	• Difficulty breathing
• लकड़ी या कंडे पर खाना बनाना	• Cooking on wood or dung cakes	• बार-बार सर्दी-जुकाम होना	• Frequent colds
ध्यान रखें — यह प्रदूषण दिखता नहीं, पर आपकी सेहत पर असर डालता है!	Remember — this pollution is invisible, but it affects your health!	• थकान और कमज़ोरी	• Fatigue and weakness
		• अस्थमा	• Asthma
		• फेफड़ों की बीमारियाँ	• Lung diseases
		• हार्ट अटैक	• Heart attack
		• स्ट्रोक	• Stroke
		• लंग कैंसर (फेफड़ों का कैंसर)	• Lung cancer
		घर के अंदर की हवा, आपकी सेहत की चाबी!	The air inside your home is the key to your health!
		अधिक जानकारी और सलाह के लिए अपने नज़दीकी स्वास्थ्य केंद्र से संपर्क करें।	For more information and advice, contact your nearest health centre.

**Table 3:** English translation of Hindi text in Figure 4 Poster 1 (left) covers sources of indoor air pollution and behaviour change messages. Poster 2 (right) covers vulnerable groups, symptoms, and health effects. Poster 1 replaces the sources list with the behaviour change section using ✗/✓ icons. Font size increased and pictograms added.

Poster 1 (left)		Poster 2 (right)	
Hindi text	English translation	Hindi text	English translation
क्या आप जानते हैं?	Did you know?	वायु प्रदूषण	Air Pollution
घर में भी हो सकता है वायु प्रदूषण!	Air pollution can happen inside your home too!	खतरा बाहर ही नहीं, आपके घर के अंदर भी है!	The danger is not just outside — it is inside your home too!
क्या आपकी रसोई में खिड़की है?	Does your kitchen have a window?	छोटे बच्चे	Small children
क्या आप उसे बंद रखते हैं?	Do you keep it closed?	गर्भवती महिलाएं	Pregnant women
घर में एग्ज़ॉस्ट फैन या चिमनी नहीं है?	Is there no exhaust fan or chimney at home?	बुज़ुर्ग लोग	Elderly people
क्या आपके घर में कोई सिगरेट पीता है?	Does anyone in your house smoke?	अस्थमा, साँस की बीमारी वाले मरीज	Patients with asthma and respiratory diseases
या मच्छर की कॉयल जलाते है?	Or burn mosquito coils?	दिल की बीमारी वाले	People with heart disease
अगर हाँ, तो आप इनडोर एयर पॉल्यूशन यानी घर के अंदर वायु प्रदूषण के संपर्क में हैं।	If yes, you are exposed to indoor air pollution.	किन लोगों को सबसे ज़्यादा खतरा है?	Who is most at risk?
घर के अंदर की हवा को बेहतर बनाए रखने के लिए इन बातों का ध्यान रखें:	Keep these things in mind to improve the air inside your home:	⚠️क्या लक्षण दिखते हैं? कौन-कौन सी बीमारियाँ हो सकती हैं?	⚠️ What symptoms appear? What diseases can occur?
❌ लकड़ी या कंडे का प्रयोग न करें ✔️	❌ Do not use wood or dung cakes ✔️	• बार-बार खाँसी आना	• Frequent coughing
❌ अगर LPG खत्म हो जाए, तो कोशिश करें कि खाना खुले या हवादार जगह पर बनाएं। ✔️	❌ If LPG runs out, try to cook in an open or well-ventilated area ✔️	• गले में जलन या खराश	• Burning or soreness in the throat
❌ खाना बनाते समय खिड़कियाँ खोलें ✔️	❌ Open windows while cooking ✔️	• साँस लेने में तकलीफ	• Difficulty breathing
❌ ताकि धुआँ और गर्मी बाहर निकल सके। ✔️	❌ So that smoke and heat can escape ✔️	• बार-बार सर्दी-जुकाम होना	• Frequent colds
❌ यदि संभव हो तो रसोई में एग्ज़ॉस्ट फैन या चिमनी लगवाएं ✔️	❌ If possible, install an exhaust fan or chimney in the kitchen ✔️	• थकान और कमज़ोरी	• Fatigue and weakness
❌ इससे धुआँ और गैस बाहर निकलती है। ✔️	❌ This helps smoke and gas escape ✔️	• अस्थमा / दमा	• Asthma
❌ सिगरेट न पिएं — घर के अंदर तो बिल्कुल भी नहीं। ✔️	❌ Do not smoke — especially not inside the house ✔️	• फेफड़ों की बीमारियाँ	• Lung diseases
❌ इससे घर के सभी लोगों को नुकसान हो सकता है। अगरबत्ती और मच्छर की कॉयल का सीमित उपयोग करें, और खिड़की खोलकर जलाएं।✔️	❌ This can harm everyone in the household Use incense sticks and mosquito coils sparingly, and burn them near an open window✔️	• हार्ट अटैक / दिल का दौरा	• Heart attack
❌ घर के अंदर मनीप्लांट जैसे पौधे लगाएं ✔️	❌ Plant indoor plants like money plant ✔️	• स्ट्रोक / लकवा	• Stroke / paralysis
❌ ये हवा को शुद्ध करने में मदद करते हैं। ✔️	❌ These help purify the air ✔️	• लंग कैंसर / फेफड़ों का कैंसर	• Lung cancer
ध्यान रखें — यह प्रदूषण दिखता नहीं, पर आपकी सेहत पर असर डालता है!	Remember — this pollution is invisible, but it affects your health!	घर के अंदर की हवा, आपकी सेहत की चाबी!	The air inside your home is the key to your health!
		अधिक जानकारी और सलाह के लिए अपने नज़दीकी स्वास्थ्य केंद्र से संपर्क करें।	For more information and advice, contact your nearest health centre.

**Table 4:** English translation of Hindi text in Figure 5 Poster 1 (left) covers sources of indoor air pollution and behaviour change messages. Poster 2 (right) covers vulnerable groups, symptoms, and health effects. Further simplification of sentence structure, improved spacing, and minimum 16-point Hindi font throughout. SMOG grade of final text: 8.1.

Poster 1 (left)		Poster 2 (right)	
Hindi text	English translation	Hindi text	English translation
क्या आप जानते हैं?	Did you know?	वायु प्रदूषण	Air Pollution
घर में भी हो सकता है वायु प्रदूषण!	Air pollution can happen inside your home too!	खतरा बाहर ही नहीं, आपके घर के अंदर भी है!	The danger is not just outside — it is inside your home too!
क्या आपकी रसोई में खिड़की है?	Does your kitchen have a window?	छोटे बच्चे	Small children
क्या आप उसे बंद रखते हैं?	Do you keep it closed?	गर्भवती महिलाएं	Pregnant women
घर में एग्ज़ॉस्ट फैन या चिमनी नहीं है?	Is there no exhaust fan or chimney at home?	बुज़ुर्ग लोग	Elderly people
क्या आपके घर में कोई सिगरेट पीता है?	Does anyone in your house smoke?	अस्थमा, साँस की बीमारी वाले मरीज	Patients with asthma and respiratory diseases
या मच्छर की कॉयल जलाते है?	Or burn mosquito coils?	दिल की बीमारी वाले	People with heart disease
अगर हाँ, तो आप इनडोर एयर पॉल्यूशन यानी घर के अंदर वायु प्रदूषण के संपर्क में हैं।	If yes, you are exposed to indoor air pollution.	किन लोगों को सबसे ज़्यादा खतरा है?	Who is most at risk?
घर के अंदर की हवा को बेहतर बनाए रखने के लिए इन बातों का ध्यान रखें:	Keep these things in mind to improve the air inside your home:	⚠️क्या लक्षण दिखते हैं? कौन-कौन सी बीमारियाँ हो सकती हैं?	⚠️ What symptoms appear? What diseases can occur?
❌ लकड़ी या कंडे का प्रयोग न करें ✔️	❌ Do not use wood or dung cakes ✔️	• बार-बार खाँसी आना	• Frequent coughing
❌ खाना बनाते समय खिड़कियाँ खोलें ✔️	❌ Open windows while cooking ✔️	• गले में जलन या खराश	• Burning or soreness in the throat
❌ यदि संभव हो तो रसोई में एग्ज़ॉस्ट फैन या चिमनी लगवाएं ✔️	❌ If possible, install an exhaust fan or chimney in the kitchen ✔️	• साँस लेने में तकलीफ	• Difficulty breathing
❌ सिगरेट न पिएं — घर के अंदर तो बिल्कुल भी नहीं। ✔️	❌ Do not smoke — especially not inside the house ✔️	• बार-बार सर्दी-जुकाम होना	• Frequent colds
❌ घर के अंदर मनीप्लांट जैसे पौधे लगाएं ✔️	❌ Plant indoor plants like money plant ✔️	• थकान और कमज़ोरी	• Fatigue and weakness
ध्यान रखें — यह प्रदूषण दिखता नहीं, पर आपकी सेहत पर असर डालता है!	Remember — this pollution is invisible, but it affects your health!	• अस्थमा / दमा	• Asthma
		• फेफड़ों की बीमारियाँ	• Lung diseases
		• हार्ट अटैक / दिल का दौरा	• Heart attack
		• स्ट्रोक / लकवा	• Stroke / paralysis
		• लंग कैंसर / फेफड़ों का कैंसर	• Lung cancer
		घर के अंदर की हवा, आपकी सेहत की चाबी!	The air inside your home is the key to your health!
		अधिक जानकारी और सलाह के लिए अपने नज़दीकी स्वास्थ्य केंद्र से संपर्क करें।	For more information and advice, contact your nearest health centre.

Phase 4: Pilot Implementation

The final IEC posters (shown in Figure 5) were put up at Saibaba Nagar UPHC serving the study community. A health education session was conducted on July 19, 2025, for patients and attendants visiting the facility. The session was conducted in Hindi using the posters as visual aids and covered three areas: common household sources of IAP, health effects of indoor pollution, and simple steps people can take to reduce their exposure - such as opening windows while cooking, keeping mosquito coils away from sleeping areas, and avoiding supplementary biomass use indoors. Attendance was not formally recorded, as this was a feasibility-focused pilot implementation session.

## Results

Sociodemographic Profile

Thirty-two households took part in the KAP survey. Most respondents were women (78%), and the mean age was 42 years (range: 22-78). The most common level of education was higher secondary (37.5%), and around one-fifth had secondary schooling. Mean monthly household income was approximately Rs 20,000. Over half of households were nuclear families (53%), and most dwellings were semi-pucca structures (65.6%). No kuccha dwellings were recorded. Full details are in Table 5.

**Table 5:** Sociodemographic characteristics of the study participants (N = 32)

Characteristic	Category	N (%)
Sex	Female	25 (78.1%)
	Male	7 (21.9%)
Age (years)	Mean (range)	42 (22-78)
Education	No formal education	4 (12.5%)
	Primary	3 (9.4%)
	Secondary	7 (21.9%)
	Higher secondary	12 (37.5%)
	Graduate or above	6 (18.8%)
Monthly household income	Mean	~Rs 20,000
Family type	Nuclear	17 (53.1%)
	Extended	10 (31.3%)
	Joint	5 (15.6%)
	Pucca	11 (34.4%)
	Semi-pucca	21 (65.6%)
	Kuccha	0 (0.0%)

Fuel Use, Cooking Practices, Ventilation, and Other Indoor Pollutant Sources

All 32 households used LPG as their primary cooking fuel, consistent with PMUY coverage in the area [3]. However, 18 households (56.3%) also used supplementary solid fuels at times, most commonly because the LPG cylinder had run out. More than half of households (56.3%) cooked in the living room with no separate kitchen. Ventilation was poor across the board - 94% of cooking areas had no chimney or exhaust fan, and 53% had no window at all. Field observation showed a structural problem common to row-house construction in this locality: windows were only at the front of the house, while cooking took place at the back, so opening windows did little to clear cooking smoke. Daily use of incense sticks or mosquito coils was reported by 75% of respondents; not a single household reported never using these products. Burning of these items is a recognised source of PM2.5 and volatile organic compounds [12]. All findings on fuel use, cooking, ventilation, and other pollutant sources are summarised in Table 6.

**Table 6:** Fuel use, cooking practices, ventilation, and other indoor pollutant sources (N = 32) *Percentages among 18 households reporting supplementary fuel use. **Percentages among 15 households with at least one window in the cooking area; responses were not mutually exclusive. LPG: liquefied petroleum gas; PM2.5: fine particulate matter ≤2.5 µm. Shaded rows denote subheadings.

Variable	Category/Response	N (%)
LPG as primary cooking fuel	Yes	32 (100.0%)
Supplementary solid fuel use	Yes	18 (56.3%)
Supplementary fuel use - reason	LPG cylinder exhaustion	14 (77.8%)*
Supplementary fuel use - reason	Taste preference (traditional chulha)	3 (16.7%)*
Supplementary fuel use - reason	Heating water in winter (biomass)	1 (5.6%)*
Cooking location	Living room - no separate kitchen	18 (56.3%)
Cooking location	Separate kitchen	14 (43.8%)
Cooking location	Outdoors	0 (0.0%)
Chimney or exhaust fan in the cooking area	Absent	30 (93.8%)
Chimney or exhaust fan in the cooking area	Present	2 (6.3%)
Window in the cooking area	Absent	17 (53.1%)
Window in cooking area	Present	15 (46.9%)
Window use pattern (among households with windows, n=15)	Opened only while cooking	14 (93.3%)**
Window use pattern (among households with windows, n=15)	Always open	3 (20.0%)**
Window use pattern (among households with windows, n=15)	Opened in the evening only	2 (13.3%)**
Window use pattern (among households with windows, n=15)	Kept closed - fear of mosquitoes	3 (20.0%)**
Indoor smoking in the household	Yes	6 (18.8%)
Incense sticks or mosquito coil use	Daily	24 (75.0%)
Incense sticks or mosquito coil use	Weekly	6 (18.8%)
Incense sticks or mosquito coil use	Occasional	2 (6.3%)
Incense sticks or mosquito coil use	None	0 (0.0%)

Knowledge, Perceptions, and Attitudes

Nearly two-thirds of respondents (62.5%, 20/32) had never heard of indoor air pollution as a concept. Among the 12 who had some awareness, almost all framed it as outdoor vehicle smoke. Three respondents specifically said they had no air pollution at home because they used LPG - a common but incorrect belief that using clean fuel alone eliminates all indoor pollution risk [4,5]. No respondent spontaneously mentioned cardiovascular, neurological, or developmental effects of IAP; awareness of health consequences was limited to cough and lung problems. When asked whether their home was affected by air pollution, common answers were "we keep our house clean" or "we only use LPG." Most respondents (78.1%) did not feel any changes to their household practices were needed. These findings - that people see little risk and no need to act - are consistent with the Health Belief Model's constructs of low perceived susceptibility and high perceived barriers [10], and directly shaped the IEC development approach. IEC content was focused on low-cost, immediately doable steps rather than structural changes, in line with self-efficacy principles [13]. Full knowledge, perception, and attitude data are in Table 7. 

**Table 7:** Knowledge, perceptions, and attitudes regarding indoor air pollution (N = 32) IAP: indoor air pollution; LPG: liquefied petroleum gas Shaded rows denote subheadings. Multiple responses were allowed for knowledge items.

Item	Response	N (%)
Awareness of IAP	Never heard of/could not define IAP	20 (62.5%)
Awareness of IAP	Some awareness of IAP (any concept)	12 (37.5%)
Description of air pollution (among aware respondents)	Outdoor vehicle smoke only	7 (21.9%)
Description of air pollution (among aware respondents)	Bathroom odour	1 (3.1%)
Description of air pollution (among aware respondents)	Cigarette or vehicle smoke	1 (3.1%)
Description of air pollution (among aware respondents)	Cited LPG use as proof of no indoor pollution	3 (9.4%)
Knowledge of health effects of IAP	Aware of any health effects	12 (37.5%)
Knowledge of health effects of IAP	Mentioned respiratory effects only (cough, lung problems)	12 (37.5%)
Knowledge of health effects of IAP	Spontaneously mentioned cardiovascular or neurological effects	0 (0.0%)
Perception of own home	Perceived home as pollution-free ("we keep our house clean" or "we only use LPG")	Common response
Willingness to change household practices	Did not consider any change necessary	25 (78.1%)
Willingness to change household practices	Felt awareness alone would motivate change	5 (15.6%)
Willingness to change household practices	Interested in structural modifications with subsidy support	2 (6.3%)

Review of Existing IEC Materials

IEC materials were retrieved from the NCDC, DGHS Delhi, and NCAP [9]. None of the reviewed materials covered household-level IAP sources, such as incense sticks, mosquito coils, solid fuel cooking, or poor ventilation - the focus was entirely on outdoor ambient pollution. No material was available in plain, locally contextualised Hindi. The SMOG grade of the NCDC English-language poster was 10.2 [6], which is above a 10th-grade reading level and well beyond the reach of most of the study population. Examples of the reviewed materials are shown in Figure 1. These findings from the document review, combined with the KAP survey results, confirmed the need for locally developed, Hindi-language IEC materials addressing household-level IAP.

IEC Material Development and Readability

Two Hindi-language posters were developed: Poster 1 on household sources of IAP and practical prevention steps, and Poster 2 on who is most at risk and what health problems IAP can cause. After two rounds of community feedback using the teach-back method [7], font size was increased, sentence structure was simplified, pictures were added alongside each behaviour change message, and sections were better separated. The ✗/✓ icon format - showing wrong practice on the left and the correct alternative on the right - was introduced in response to community preference for clear, actionable guidance. The SMOG grade improved from 7.9 (initial draft) to 8.1 (final version), compared to 10.2 for the NCDC reference material (Table 8). The poster development process across all three versions is shown in Figures 3-5.

**Table 8:** SMOG readability scores of IEC materials SMOG: Simple Measure of Gobbledygook; NCDC: National Centre for Disease Control A lower SMOG score indicates greater readability.

IEC Material	Language	SMOG Grade
NCDC existing poster (reference)	English	10.2
Study poster - initial draft	Hindi	7.9
Study poster - revised final version	Hindi	8.1

Pilot Implementation

The final IEC posters (shown in Figure 5) were displayed at Saibaba Nagar UPHC. A health education session was conducted on July 19, 2025, for patients and attendants visiting the facility. The session was conducted in Hindi using the posters as visual aids and covered: common household sources of IAP (poor ventilation, supplementary solid fuels, incense and mosquito coil use, indoor smoking), the health effects of IAP, and specific practical steps - opening windows while cooking, keeping mosquito coils near doors rather than in sleeping or cooking areas, avoiding biomass indoors when LPG is unavailable, and fitting an exhaust fan in the kitchen if possible.

## Discussion

Despite near-universal LPG use, indoor air pollution remained a significant problem in this urban slum community. Supplementary solid fuel use, cooking in the living room, no exhaust ventilation, and daily burning of incense sticks and mosquito coils all contributed to continued indoor pollutant exposure even in households that had switched to clean fuel. This is in line with findings from other studies in post-Ujjwala settings, which show that fuel substitution alone is not enough to eliminate IAP [4].

The most important finding from the KAP survey was that 62.5% of respondents had never heard of indoor air pollution as a distinct concept. This pattern has been reported elsewhere - Priyadarsini et al. found similar awareness gaps in urban slum populations in Tamil Nadu [5], and Maharana et al. identified poor IAP awareness among women in Kolkata slums despite high household exposure [6]. The fact that three respondents in our study cited LPG use as proof that their home had no air pollution shows how specific and persistent this misconception is. Health communication in the post-LPG transition period needs to explicitly address IAP from secondary sources - incense, mosquito coils, supplementary biomass, and poor ventilation - rather than simply promoting fuel switching.

Ventilation findings deserve particular attention. Not only did 94% of cooking areas lack a chimney or exhaust fan, but field observations identified a structural problem that is unlikely to be picked up by a questionnaire alone: in the row-house construction typical of this locality, windows are located at the front of the house while cooking happens at the back. This means that the standard IEC advice to "open windows while cooking" is physically impractical for most households here. This finding supports the case for community-level structural observation as part of IEC development, rather than using generic messages that assume all homes are built the same way.

The prevalence of daily incense stick and mosquito coil use (75%) was high and is an underappreciated IAP risk in this setting. Burning of these products indoors generates PM2.5, volatile organic compounds, and polycyclic aromatic hydrocarbons at concentrations that can substantially exceed safe limits in poorly ventilated spaces [13]. Despite this, neither incense nor mosquito coils featured in any of the existing national IEC materials reviewed. Including specific, practical guidance on these products - such as burning incense near an open door rather than in a closed bedroom, and placing mosquito coils at floor level near an exit - is a low-cost, actionable message that is directly relevant to this population.

The IEC messages in this study were deliberately restricted to actions feasible within existing structural constraints, recognising that education alone cannot address the ventilation deficits inherent to row-house construction in this setting. Structural interventions require policy-level action beyond the scope of health communication.

The readability findings strengthen the case for community-based IEC development. The final revised poster had an SMOG grade of 8.1 compared to 10.2 for the NCDC poster [7], which is above a 10th-grade reading level. A SMOG grade of 8 corresponds roughly to middle-school level - still not simple, but a meaningful step closer to the literacy level of the study population. This gap between what is produced nationally and what communities can actually read has been noted by health communication researchers [8]. The teach-back process [8] was useful not just for checking comprehension but for getting specific, practical design feedback - participants pointed out exactly which sentences were hard and which pictures helped. This kind of direct community input is difficult to replicate through expert review alone.

The attitude data - 78% of respondents seeing no need to change anything - fits the Health Belief Model's explanation of why people do not act even when they face a health risk: low perceived susceptibility and high perceived barriers [10]. The IEC approach was designed with this in mind. Recommended actions were kept simple, cheap, and immediately doable, without requiring structural changes or extra spending. This approach draws on self-efficacy theory [14], which holds that people are more likely to change behaviour when they feel capable of doing so. Telling someone to install an exhaust fan is not a feasible message for most households in this setting; telling someone to open the front door while burning incense is.

Future studies should also explore the specific socioeconomic, structural, and cultural determinants of the resistance to behaviour change observed in this community - including the 78.1% who saw no need to change - through qualitative methods such as in-depth interviews or focus group discussions. Scaled delivery through Accredited Social Health Activist (ASHA) and Auxiliary Nurse Midwife (ANM) networks, combined with structured outcome evaluation, represents the logical next step toward assessing whether these materials can drive sustained behaviour change across larger, more diverse urban populations.

Limitations

This activity had a small sample of 32 households in a single locality, which limits how widely the findings can be applied. Convenience sampling may have favoured households that were easier to reach. There was no formal pre- and post-test of knowledge or attitudes, so we cannot measure how much the health talks changed awareness. The health education session in Phase 4 was conducted at the UPHC and may not have reached the same households that participated in the Phase 1 KAP survey - this population mismatch between the needs assessment and implementation phases is a limitation of this pilot activity. The KAP questionnaire was developed de novo and underwent face and content validity checks through expert review and pilot testing; however, formal psychometric validation and reliability testing were not conducted. The pilot implementation was conducted as a single health education session without formal outcome evaluation. Attendance was not formally recorded, and no pre-post knowledge or behaviour assessment was performed, which limits conclusions about the immediate impact of the IEC materials on participant awareness or behaviour. Indoor PM2.5 levels were not objectively measured at any stage of this activity, and it is therefore not possible to determine whether the IEC intervention translated into any reduction in actual pollutant exposure in the home. Future work in this area should include a pre-post KAP design, a larger sample across multiple localities using systematic random sampling, and objective measurement of indoor PM2.5 levels to link health promotion activities to actual exposure changes. Additionally, longitudinal follow-up assessments, inclusion of home-based businesses as settings with unique pollutant sources, and structured outcome evaluation, including attendance recording and participant feedback, should be incorporated.

## Conclusions

This pilot activity shows that a community needs assessment followed by participatory IEC development is both practical and useful in an urban slum setting. The KAP survey uncovered specific knowledge gaps and structural factors - particularly around incense and mosquito coil use, supplementary fuel burning, and the physical layout of row-house kitchens - that were not reflected in any existing national IEC material. The Hindi-language posters developed through this process were more readable and more relevant to this community than the available reference materials. The SMOG grade of the final poster (8.1) represented a meaningful improvement over the national reference material (10.2), and the materials were delivered through a health education session at Saibaba Nagar UPHC. A community needs assessment before developing IEC materials, rather than adapting existing national content, should be standard practice in local environmental health programmes. If delivered through ASHA and ANM networks, this kind of targeted, readable, locally grounded IEC represents a feasible first step toward the broader goal of reducing IAP in households that have adopted clean fuels but continue to face significant indoor pollutant exposures.

## Appendices

KAP Questionnaire on Indoor Air Pollution

घर के अंदर वायु प्रदूषण पर KAP प्रश्नावली

Department of Community and Family Medicine, AIIMS Bhopal

Urban Slum, Ward 49, Bhopal

Participant ID: _____________   Date: _____________   Interviewer: _____________

Section A: Sociodemographic Information  |  भाग क: सामाजिक-जनसांख्यिकीय जानकारी

1. Name of respondent:  _______________________  (उत्तरदाता का नाम)

2. Age (years):  _______________________  (आयु (वर्ष))

3. Sex  / लिंग

☐  Male / पुरुष  / 

☐  Female / महिला  / 

4. Education level  / शिक्षा स्तर

☐  Illiterate / निरक्षर  / 

☐  Primary / प्राथमिक  / 

☐  Secondary / माध्यमिक  / 

☐  Higher Secondary / उच्चतर माध्यमिक  / 

☐  Graduate / स्नातक  / 

☐  Post Graduate / स्नातकोत्तर  / 

5. Occupation:  _______________________  (व्यवसाय)

6. Average monthly household income (Rs):  _______________________  (औसत मासिक आय (रु.))

7. Type of family  / परिवार का प्रकार

☐  Nuclear / एकल परिवार  / 

☐  Joint / संयुक्त परिवार  / 

☐  Extended / विस्तारित परिवार  / 

8. Type of house  / घर का प्रकार

☐  Pucca / पक्का  / 

☐  Semi-pucca / अर्ध-पक्का  / 

☐  Kuccha / कच्चा  / 

9. Number of rooms:  _______________________  (कमरों की संख्या)

10. Number of family members:  _______________________  (परिवार के सदस्यों की संख्या)

Section B: Daily Household Practices  |  भाग ख: घरेलू आदतें

1. What fuel do you mainly use for cooking?  / आप खाना बनाने के लिए मुख्य रूप से कौन सा ईंधन इस्तेमाल करते हैं?

☐  LPG / एलपीजी  / 

☐  Kerosene / मिट्टी का तेल  / 

☐  Firewood / लकड़ी  / 

☐  Dung cake / कंडा  / 

Other / अन्य:  ___________________________  /  :  ___________________________

2. Do you ever use any other fuel for cooking or heating, even sometimes?  / क्या आप कभी-कभी खाना बनाने या हीटर जलाने के लिए कोई और ईंधन भी इस्तेमाल करते हैं?

☐  Yes / हाँ  / 

☐  No / नहीं  / 

If yes, which one / यदि हाँ, तो कौन सा:  ___________________________  /  :  ___________________________

3. Where do you usually cook food?  / आप आमतौर पर कहाँ खाना बनाते हैं?

☐  Separate kitchen / अलग रसोई  / 

☐  Living or sleeping area / कमरे / सोने का कमरा  / 

☐  Veranda or outside / बरामदा / बाहर  / 

4. Are there any windows or openings in your kitchen?  / क्या आपकी रसोई में खिड़कियाँ या रोशनदान हैं?

☐  Yes / हाँ  / 

☐  No / नहीं  / 

If yes, when do you keep them open / यदि हाँ, तो आप इन्हें कब खुला रखते हैं:  ___________________________  /  :  ___________________________

5. Do you have a fan, vent, or chimney in the cooking area?  / क्या आपकी रसोई में पंखा, वेंट या चिमनी है?

☐  Yes / हाँ  / 

☐  No / नहीं  / 

6. Does anyone in your household smoke?  / क्या आपके घर में कोई धूम्रपान करता है?

☐  Yes / हाँ  / 

☐  No / नहीं  / 

If yes, where do they usually smoke / यदि हाँ, तो वे कहाँ धूम्रपान करते हैं:  ___________________________  /  :  ___________________________

7. Do you or anyone in your family burn incense sticks, mosquito coils, or similar items at home?  / क्या आप या आपके परिवार में कोई अगरबत्ती, मच्छर कॉइल या ऐसा कुछ जलाते हैं?

☐  Yes / हाँ  / 

☐  No / नहीं  / 

If yes, how often?  / यदि हाँ, तो कितनी बार?

☐  Daily / रोज़ाना  / 

☐  Weekly / साप्ताहिक  / 

☐  Occasionally / कभी-कभी  / 

Section C: Knowledge and Beliefs  |  भाग ग: ज्ञान और विचार

8. What comes to your mind when you hear the term "air pollution"?  / जब आप "वायु प्रदूषण" शब्द सुनते हैं तो आपके मन में क्या आता है?

____________________________________________________________

____________________________________________________________

9. What do you think could cause pollution inside the house?  / आपके अनुसार घर के अंदर प्रदूषण के क्या कारण हो सकते हैं?

____________________________________________________________

____________________________________________________________

10. Have you heard of any health problems related to air inside the home? If yes, which ones?  / क्या आपने घर के अंदर की हवा से होने वाली किसी बीमारी के बारे में सुना है? यदि हाँ, तो कौन-कौन सी?

☐  Yes / हाँ  / 

☐  No / नहीं  / 

If yes, specify / यदि हाँ, तो बताएं:  ___________________________  /  :  ___________________________

11. Do you feel there is any smoke or bad air inside your house? If yes, when or where do you feel it most?  / क्या आपको लगता है कि आपके घर में कभी धुआं या खराब हवा होती है? यदि हाँ, तो कब या कहाँ ज़्यादा महसूस होता है?

☐  Yes / हाँ  / 

☐  No / नहीं  / 

If yes, when or where / यदि हाँ, तो कब और कहाँ:  ___________________________  /  :  ___________________________

Section D: Possible Changes  |  भाग घ: संभावित बदलाव

12. What support would help you keep your house well ventilated?  / आपको अपना घर हवादार रखने के लिए किस तरह की मदद चाहिए?

☐  Structural improvement / घर की मरम्मत  / 

☐  Subsidy for exhaust fan / एग्ज़ॉस्ट पंखे के लिए मदद  / 

☐  Awareness only / सिर्फ जागरूकता  / 

Others / अन्य:  ___________________________  /  :  ___________________________

13. If you wanted to make your house smoke-free, what challenges might you face?  / अगर आप घर को धुएँ से मुक्त बनाना चाहें तो आपको क्या-क्या मुश्किलें आ सकती हैं?

____________________________________________________________

____________________________________________________________

14. Any other comments or suggestions:  / कोई और राय या सुझाव:

____________________________________________________________

____________________________________________________________

Thank you for your participation. / आपकी भागीदारी के लिए धन्यवाद।
